# Robot-assisted laser interstitial thermal therapy for drug-resistant epilepsy in hypothalamic hamartoma

**DOI:** 10.3171/2024.4.FOCVID2415

**Published:** 2024-07-01

**Authors:** James Bridges, Michael Folse, Danielle Terrell, Jehan Bista, Jamie Toms

**Affiliations:** 1 School of Medicine, LSU Health Shreveport, Louisiana;; 2 Department of Neurosurgery, LSU Health Shreveport, Louisiana; and; 3 School of Medicine, The University of Queensland Ochsner Clinical School, New Orleans, Louisiana

**Keywords:** LITT, hypothalamic hamartoma, drug-resistant epilepsy, stereotactic biopsy

## Abstract

Hypothalamic hamartomas are congenital lesions of the hypothalamus, with a range of symptoms defined by lesion location. Common presenting symptoms include gelastic seizures and precocious puberty. When hamartoma-related seizures become resistant to medications, laser interstitial thermal therapy (LITT) has been shown to be an effective treatment. The authors present a case of robot-assisted LITT for a patient with an 11-year history of epilepsy due to hypothalamic hamartoma. In addition, they demonstrate the use of a stereotactic biopsy needle implemented during the procedure for possible biopsy of deep cranial lesions.

The video can be found here: https://stream.cadmore.media/r10.3171/2024.4.FOCVID2415

## Transcript

In this video, we highlight the operative steps for a robot-assisted laser interstitial thermal therapy (LITT) in the treatment of drug-resistant epilepsy caused by a hypothalamic hamartoma with concurrent use of a stereotactic biopsy needle.

### 0:36 Background.

A hypothalamic hamartoma is a congenital, nonprogressive lesion of the hypothalamus formed during fetal development.^1,2^ There are two anatomical variants of these lesions: parahypothalamic hamartomas, which are often associated with precocious puberty, and intrahypothalamic hamartomas, which are often associated with gelastic seizures, other forms of epilepsy, cognitive impairment, and psychiatric symptoms.^1^ Up to 40% of patients, however, will present with symptoms from both anatomical variations.^1^

Stereotactic laser interstitial thermal therapy, also known as LITT therapy, is an emerging, minimally invasive neurosurgical technique primarily used in the treatment of brain tumors, radiation necrosis, and epileptic foci.^1,3^ Despite first being introduced in 1983, its initial use was limited due to an inability to monitor tissue temperature and control the extent of ablation.^4^ Recent advancements in intraoperative MRI, however, have allowed for improved thermal monitoring and thus increased treatment accuracy.^3^

Previous studies have shown LITT to be an effective treatment for drug-resistant epilepsy in hypothalamic hamartomas, with as high as 80% of patients reporting seizure-free outcomes 6 months after their procedure.^5^ There currently exists limited data on risks and potential complications; however, previous studies have shown a rate of severe complications requiring medical interventions to be approximately 3%.^6^ Of these complications, weakness is reported to be the most common.^6^ Other reported complications noted to be aware of have been a worsening of diabetes insipidus and potential gadolinium extravasation into nearby ventricles.^6^

### 2:19 Case Presentation.

A 24-year-old female referred to our clinic for potential resection of her known hypothalamic mass causing a long-standing history of epileptic episodes. She reported approximately 3 gelastic seizures every month despite being on maximum antiepileptic drug therapy. These episodes typically last 30 seconds and are associated with oral automatisms and postictal confusion. Additionally, she reported being diagnosed with central precocious puberty around the age of 1, which was suppressed until the age of 12 with leuprolide. Her neurological exam was fully intact. Given these findings, in context of her known hypothalamic mass, we determined that she was an appropriate candidate for LITT therapy.

### 3:03 Preoperative Imaging.

Preoperative, T1-weighted MRI was completed, which showed a redemonstration of her known, nonenhancing mass arising from the right hypothalamus. This mass measured approximately 8 × 8 × 8 mm. Within this mass there were no areas of restricted diffusion seen and no abnormal contrast enhancement. There was no mass effect on surrounding structures, no extra-axial fluid collections, and all major blood vessels demonstrated adequate flow-related signal. In total, there were no significant changes seen when compared to previous images.

### 3:39 Positioning and Technique.

Once in the operating room and anesthetized, the patient was positioned supine and pinned with the Mayfield skull clamp. For this procedure, we used the Globus ExcelsiusGPS robotic navigation system with concurrent C-arm fluoroscopy registration. After the robot was registered with fluoroscopy, the patient is prepped, draped, and the area is marked.

### 4:02 Laser Trajectory.

Using the patient’s images, a trajectory is planned with a goal of taking the most direct path toward the hypothalamus, being sure to avoid the fornices, optic apparatus, and sulci, which may contain easily damaged blood vessels.

### 4:16 Reference Arc Placement and Robot Trajectory Placement.

The sterile reference arc is positioned adjacent to the patient’s head, and the robot is then brought into the field and guided to its planned trajectory.

### 4:25 Stab Incision.

A stab incision is made on trajectory through the End Effector arm of the robot.

### 4:31 Drilling Hole.

The reducing tube was then placed, and a hole was drilled on trajectory with high-speed burr.

### 4:39 Opening the Dura.

Opening the dura can then be confirmed using a sharp stylet. If the dura was not opened using the drilling, it can now be done.

### 5:03 Anchor Bolt Placement.

An anchor bolt is placed through the burr hole and on trajectory. The bolt is tightened 7 to 8 full turns. It may be beneficial, however, to perform 16 half-turns. You will know you remained on trajectory if the bolt is easily removed without resistance at the end.

### 5:30 Biopsy Needle Measurements.

As an alternative to using a measured stylet, we chose to use a stereotactic navigated biopsy needle to ensure the proper trajectory. At this point, if biopsy were needed, this navigated biopsy needle can be used to sample specimen.

### 5:44 Biopsy Needle Insertion.

The biopsy needle is then placed on trajectory using real-time navigation. If the trajectory is correct, the needle should be able to pass through the bolt without any resistance.

### 5:55 Removing the Robot.

The robot is then removed from the field.

### 6:00 Measurement With Navigated Probe.

A navigated probe is placed at the top of the bolt to measure the distance from the target. A sterile paper ruler can be placed over the opening of the anchor bolt to provide a flat surface for measurement.

### 6:10 Anchor Bolt Cap Placement.

A cap is then placed on top of the anchor bolt to ensure sterility and conserve cerebrospinal fluid.

### 6:19 Snap Cover Drape Placement.

A snap cover drape is placed and stapled to the patient’s scalp that can be opened sterilely once in the MRI suite.

### 6:27 Locating Ablation Site.

Once the patient is in the MRI suite, the laser is measured to length, passed through the machine, and attached to the anchoring bolt. A T1-weighted noncontrast sequence MRI with the laser in place is obtained to be identified by the laser software and ensure proper location.

### 6:46 Laser Ablation.

Under MRI thermography, the laser is heated to 43°C and the lesion is ablated. For this particular hamartoma, only one pass of the laser was needed. If the lesion is larger, however, multiple trajectories may be planned. Alternatively, we may choose to reposition the laser along the length of the lesion, taking particular care to not injure any vital hypothalamic structures. Once treatment is complete, a postoperative MRI is obtained, the laser and bolt are removed, and the small stab incision is closed with a single nylon figure-of-eight stitch.

### 7:21 Postoperative Imaging.

After ablation, a T1-weighted gadolinium MRI was obtained to demonstrate the postoperative changes of LITT therapy. At this point, the probe tip was still within the right hypothalamus.

### 7:36 Postoperative Outcome.

Two weeks postop, the patient denied any seizure-like activity and her neurological exam continued to be unremarkable. To assess her endocrine function, we measured her ACTH, LH, FSH, prolactin, insulin-like growth factor, TSH, free T3, and free T4 levels, all of which came back within normal limits.

### 7:59 Concluding Remarks.

This video demonstrates the use of LITT therapy as a minimally invasive method for definitive treatment of hypothalamic hamartoma. This technique, combined with recent advancements in robotic systems, can allow for a highly accurate, expedited approach to epilepsy surgery that maintains high efficacy.

Thank you for listening. Presented here are our references.

## Disclosures

Dr. Toms reported personal fees from Globus outside the submitted work.

## Author Contributions

Primary surgeon: Toms. Assistant surgeon: Terrell. Editing and drafting the video and abstract: Bridges, Folse, Terrell, Toms. Critically revising the work: all authors. Reviewed submitted version of the work: all authors. Approved the final version of the work on behalf of all authors: Bridges. Supervision: Bridges, Toms.

## Supplemental Information

### Patient Informed Consent

The necessary patient informed consent was obtained in this study.
